# Optimization of contrast medium volume for abdominal CT in oncologic patients: prospective comparison between fixed and lean body weight-adapted dosing protocols

**DOI:** 10.1186/s13244-021-00980-0

**Published:** 2021-03-20

**Authors:** Damiano Caruso, Elisa Rosati, Nicola Panvini, Marco Rengo, Davide Bellini, Giulia Moltoni, Benedetta Bracci, Elena Lucertini, Marta Zerunian, Michela Polici, Domenico De Santis, Elsa Iannicelli, Paolo Anibaldi, Iacopo Carbone, Andrea Laghi

**Affiliations:** 1 Radiology Unit, Department of Surgical and Medical Sciences and Translational Medicine, Sapienza University of Rome - Sant’Andrea University Hospital, Via di Grottarossa, 1035-1039, 00189 Rome, Italy; 2 Diagnostic Imaging Unit, Department of Medico-Surgical Sciences and Biotechnologies, ICOT Hospital, “Sapienza” University of Rome, Via Franco Faggiana, 1668, 04100 Latina, Italy; 3 Hospital Direction and Clinical Departments, Sant’Andrea University Hospital, Via di Grottarossa, 1035-1039, 00189 Rome, Italy

**Keywords:** Computed tomography, Contrast media, Lean body weight, Oncologic imaging, Abdomen

## Abstract

**Background:**

Patient body size represents the main determinant of parenchymal enhancement and by adjusting the contrast media (CM) dose to patient weight may be a more appropriate approach to avoid a patient over dosage of CM. To compare the performance of fixed-dose and lean body weight (LBW)-adapted contrast media dosing protocols, in terms of image quality and parenchymal enhancement.

**Results:**

One-hundred cancer patients undergoing multiphasic abdominal CT were prospectively enrolled in this multicentric study and randomly divided in two groups: patients in fixed-dose group (*n* = 50) received 120 mL of CM while in LBW group (*n* = 50) the amount of CM was computed according to the patient’s LBW. LBW protocol group received a significantly lower amount of CM (103.47 ± 17.65 mL vs. 120.00 ± 0.00 mL, *p* < 0.001). Arterial kidney signal-to-noise ratio (SNR) and contrast-to-noise ratio (CNR) and pancreatic CNR were significantly higher in LBW group (all *p* ≤ 0.004). LBW group provided significantly higher arterial liver, kidney, and pancreatic contrast enhancement index (CEI) and portal venous phase kidney CEI (all *p* ≤ 0.002). Significantly lower portal vein SNR and CNR were observed in LBW-Group (all *p* ≤ 0.020).

**Conclusions:**

LBW-adapted CM administration for abdominal CT reduces the volume of injected CM and improves both image quality and parenchymal enhancement.

## Key points

- Lean body weight (LBW) group received significantly lower amount of contrast media.
- Higher arterial contrast enhancement index (CEI) and kidney portal-venous CEI in LBW-group.
- LBW protocol may be considered for routine to improve CT performance.

## Background

Contrast-enhanced CT is the imaging modality of choice for the study of the abdomen in a large spectrum of diseases. Patient body size is known to be a major determinant of parenchymal enhancement, and it has been shown that adjusting the contrast media (CM) dose to patient weight may be a more appropriate approach than administering a fixed dose of CM, allowing to reduce inter-patient variability and unnecessary healthcare costs related to CM over dosage [1–6]. However, there are no general recommendations or established guidelines regarding the CM dosing method necessary to optimize parenchymal enhancement for abdominopelvic CT studies, and fixed-dose CM injection protocols are still commonly used for clinical practice in many institutions [7, 8].

Due this lack of standardization, multiple weight-adapted CM dosing regimens have been proposed, based on a large variety of body size indexes, including total body weight (TBW), body mass index (BMI), lean body weight (LBW), and body surface area (BSA), but no consensus on has been reached so far [9–13].

Dosing CM according to patient TBW does not require complicated calculations and, given its quickness and ease of use, can be readily implemented in the daily routine; nonetheless, it fails to take into account differences in body composition. In particular, TBW-adapted regimens may lead to an overestimation of CM volume in overweight and obese patients, in which scarcely perfused adipose tissue contributes for a considerable proportion of body weight [14, 15]. Differences in body fat percentage between men and women may also result in excessive amount of CM being administered to women, when dosing CM according to TBW [16, 17].

Promising results have been reported by previous studies investigating CM dosing protocols based on LBW rather than TBW, which resulted in a reduced inter-patient variability of parenchymal and vascular enhancement [16, 18]. Tailoring CM volume to patient LBW also demonstrated to better correlate with aortic and liver enhancement compared to TBW, BSA, or blood volume [19–22]. However, the effectiveness of LBW-adapted dosing protocols has been mostly investigated exclusively on Asian population, with many studies not assessing for differences in subjective image quality.

Therefore, our aim was to perform a prospective multicenter study to compare the performance of fixed-dose and LBW-adapted CM dosing protocols for multiphasic abdominal CT, in terms of image quality and parenchymal enhancement.

## Methods

### Study population

This prospective randomized multicenter study was conducted at two centers (Sant'Andrea University Hospital, Rome, Italy; and ICOT Hospital, Latina, Italy) and was approved by the Institutional Review Board of both participating institutions. Written informed consent was obtained from all patients and the study was compliant with the Health Insurance Portability and Accountability Act. From November 2018 to March 2019 oncologic patients clinically referred for multiphasic contrast-enhanced abdominal CT were prospectively enrolled. Exclusion criteria were as follows: (a) age < 18 years; (b) history of allergic reactions to iodinated CM; (c) kidney failure (estimated glomerular filtration rate < 30 mL/min/1.73 m^2^); (d) pregnancy; (e*)* deviation from injection and acquisition protocol and (f) presence of image artifacts deemed to impair quantitative measurement. Data on patient age, gender, height, primary cancer, TBW, LBW and BMI were recorded for all participants.

### Contrast media injection protocol

Iomeprol with an iodine concentration of 350 mgI/mL (Iomeron 350; Bracco Imaging, Italy), was intravenously injected at a fixed flow rate of 3.0 mL/s (Iodine delivery rate of 1.05 gI/s) through an 18-gauge antecubital access by using an automated dual-syringe power injector (Stellant D; Medrad Inc, Warrendale, PA) and followed by a 50 mL saline chaser administered at the same flow rate.

Patients were randomly assigned into one of two CM dosing protocols using a randomization list on a 1:1 basis. Patients in the fixed dose protocol group received a fixed CM dose of 120 mL [42 g of iodine (gI) ]. Patients in the LBW protocol group received 0.7 gI per kg of LBW [10]. The resulting value was then divided by CM concentration (mgI/mL) to obtain the correct CM volume to be administered, as follows:

$$\mathrm{CM}\, \mathrm{volume }\left(\mathrm{mL}\right)=\frac{0.7 \cdot \mathrm{LBW}}{350} \cdot 1000$$

LBW was calculated by using James formula as follows [23]:

$$\begin{aligned}\mathrm{LBW}\,\mathrm{men}&=\left(1.10 \cdot {W}\right)-128 \cdot \left[\frac{{W}^{2}}{{(100 \cdot H)}^{2}}\right]\\ \mathrm{LBW}\,\mathrm{women}&=\left(1.07 \cdot {W}\right)-148 \cdot \left[\frac{{W}^{2}}{{(100 \cdot H)}^{2}}\right]\end{aligned}$$

where *W* is the patient weight in kilograms and *H* is the patient height in meters. For each CT examination, the volume of administered CM was recorded.

### Scanning protocol and image reconstruction

Imaging was performed by using a 64-row multidetector CT scanner (Lightspeed VCT, GE Medical Systems, Waukesha, WI, USA) or with a 256-slice CT (Brilliance iCT 256, Philips Healthcare, The Netherlands), based on the reference centre. However, both in fixed dose protocol group and in the LBW protocol group the number of CT scans performed with two different CT scanners were equivalent, as reported in Table 1.

**Table 1:** Contrast media protocol and CT technical specifications

Parameter	64-row CT scanner	256-row CT scanner
CM protocol		
Fixed dose protocol	25 patients	25 patients
LBW protocol	25 patients	25 patients
Scanner parameters		
Tube voltage	120 kVp	120 kVp
Beam pitch	1.375:1	1
Detector configuration	64 rows × 0.625 mm	2 × 128 rows × 0.625 mm
* z*-axis tube current modulation	Smart mA, GE Healthcare	Dose Right ACS, Philips Healthcare
Automatic mA current modulation	200/600 mAs	200/600 mAs
Noise index	28	22 DRI, Liver DRI of + 2
Slice thickness	1.25 mm	1.25 mm
Spacing	1.25 mm	1.25 mm
Iterative reconstruction	ASiR 40%; GE Healthcare	iDose4 – level 2; Philips Healthcare

*CM* contrast media, *LBW* lean body weight, *DRI* Dose Right Index

Scanning parameters for 64-row CT have been set as follows: tube voltage, 120 kVp; beam pitch, 1.375:1; detector configuration, 64 × 0.625 mm. A *z*-axis tube current modulation (Smart mA, GE Healthcare) was applied with a noise index of 28 (min/max tube current: 200/600 mAs).

For 256-slice CT, scanning parameters were: tube voltage, 120 kVp; beam pitch, 1; detector configuration, 2 × 128 rows × 0.625 mm; tube load from 200 to 600 mAs depending on automatic mA current modulation (Dose Right ACS, Philips Healthcare), with a Dose Right Index (DRI) of 22 and a Liver DRI of + 2.

All examinations were performed with patient in supine position and in a cranio-caudal direction, from the diaphragmatic dome to the pubic symphysis. The scan delay was determined using a bolus-tracking software program (SmartPrep for GE Healthcare, BolusPro for Philips Healthcare), with the placement of a 100 HU-threshold region-of-interest (ROI) within the abdominal aorta at the level of the celiac tripod. All patients in the two centres underwent a hepatic dynamic scan, including a pre-enhanced phase, a late arterial phase acquired 16 s after reaching the threshold, and a portal venous phase acquired 70 s after reaching the threshold.

Image datasets were reconstructed at the CT scanner console with the following parameters: slice thickness of 1.25 mm and spacing of 1.25 mm. Iterative reconstruction programs, “ASiR 40%; GE Healthcare” and “iDose4—level 2; Philips Healthcare”, were applied, as vendors suggested.

### Objective image quality analysis

In both centres, a reader with at least 3 years of experience (DDS) in abdominal radiology analysed all images. Attenuation measurements in HU and standard deviation (SD) values, were obtained in axial scans, by positioning a circular ROI of approximately 1 cm^2^ in the liver (segment II, IVa and VII), renal cortex of both kidneys, pancreas, and left psoas muscle in both arterial and portal venous phase. Supplementary ROIs were placed in the suprarenal abdominal aorta on arterial phase images and in the portal vein on portal venous phase images. All measurements were performed three times at the same levels and then averaged to ensure consistency. The mean of the 3 averaged liver ROIs was obtained to define the liver HU; kidney HU was defined as the mean of right and left kidney averaged ROIs. Image noise was defined as the SD measured in a circular ROI placed in subcutaneous fat tissue.

Arterial liver, pancreatic, kidney and aortic signal-to-noise ratio (SNR) and contrast-to-noise ratio (CNR) as well as portal venous phase liver, pancreatic, kidney, and portal vein SNR and CNR were calculated as follows [12]:

$$\begin{aligned}\mathrm{SNR}&= \frac{\mathrm{HU}}{\mathrm{Noise}}\\ \mathrm{CNR }&= \frac{\mathrm{HU}-\mathrm{HU}\,\mathrm{muscle}}{\mathrm{Noise}}.\end{aligned}$$

Liver, kidney and pancreas contrast enhancement on both arterial and portal venous phase was quantified by calculating the contrast enhancement index (CEI) as follows [19]:

$${\text{CEI}} = {\text{HU}}\;{\text{enhanced}} - {\text{HU}}\,{\text{unenhanced}}{.}$$

### Subjective image quality analysis

Subjective image quality was assessed independently by two experienced abdominal radiologists (M.Z. and N.P.) at each centre. All readers were unaware of which CM dosing protocol had been used. Image datasets were primarily displayed with standard window settings for evaluation of soft tissue (width: 400 HU; level: 40 HU). However, readers were allowed to freely adjust window width and level values according to their preferences. The enhancement of the liver, kidneys, and pancreas was rated by using a 5-point Likert scale as follows: 1 = very poor; 2 = poor; 3 = fair; 4 = good; 5 = excellent [20].

### Statistical analysis

Statistical analysis was performed by using the MedCalc5 Statistical Software version 17.9.7 (MedCalc Software bvba, Ostend, Belgium; http://www.medcalc.org; 2017). Continuous variables were expressed as mean ± SD or mean with ranges. Ordinal variables were expressed as median with ranges. Patient characteristics (BMI, age, and CM dose expressed in gI and mL), SNR, CNR, and CEI, were compared between the two groups. The Kolmogorov–Smirnov test was used to assess data distribution. For normally distributed data, Student’s *t* test was applied. In case of non-normally distributed data, Mann–Whitney *U* test was performed. The *x*^2^ test was used to calculate differences in gender and type of primary cancer between the two study groups.

Cohen’s Kappa test was used to evaluate inter-reader agreement for subjective image quality assessment using the following coefficients: *κ* ≤ 0.20, poor agreement; *κ* = 0.21–0.40, fair agreement; *κ* = 0.41–0.60, moderate agreement; *κ* = 0.61–0.80, good agreement; and *κ* = 0.81–1.0, excellent agreement [24]. A *p* value ≤ 0.05 was considered statistically significant.

## Results

### Study population

One-hundred-ten consecutive patients were prospectively included in the study, 10 individuals were excluded due to previous allergic reactions to iodinated CM (*n* = 2), kidney failure (*n* = 3), deviation from injection protocol (*n* = 2) and the presence of artifacts (*n* = 3). Therefore, 100 patients were finally enrolled in the study population: 50 patients in the fixed dose protocol group and 50 patients in the LBW protocol group. No significant differences were observed between the two groups in terms of patient age, gender, BMI, and LBW (all *p* ≥ 0.111). Detailed patient characteristics are reported in Table 2.

**Table 2:** Patients characteristics

Parameter	Fixed dose protocol (*n* = 50)	LBW protocol (*n* = 50)	*p* value
Gender			
Male	24 (48%)	23 (46%)	0.842
Female	26 (52%)	27 (54%)	
Age, years	63.76 ± 13.21 (38–92)	67.80 ± 11.91 (42–89)	0.111
Height, m	1.68 ± 0.10 (1.53–1.98)	1.65 ± 0.09 (1.48–1.83)	0.311
Weight, kg	73.70 ± 13.67 (45–103)	74.08 ± 15.61 (50–120)	0.897
BMI, kg/m^2^	25.98 ± 4.03 (17.58–36.21)	27.05 ± 5.26 (16.98–44.08)	0.256
LBW, kg	53.02 ± 9.94 (36.44–76.46)	51.73 ± 8.83 (37.90–71.78)	0.649
Contrast media			
Dose, gI	42.00 ± 0.00 (42.00–42.00)	36.21 ± 6.18 (26.53–50.24)	< 0.001
Volume, mL	120.00 ± 0.00 (120–120)	103.47 ± 17.65 (76–144)	< 0.001
Primary cancer			
Gastrointestinal	22 (44%)	19 (38%)	0.833
Hepatobiliary	6 (12%)	8 (16%)	
Genitourinary	11 (22%)	12 (24%)	
Prostate	7 (14%)	9 (18%)	
Other	4 (8%)	2 (4%)	

Data are numbers with percentages or means ± standard deviations, with ranges in parentheses

*BMI* body mass index, *LBW* lean body weight

### Contrast media dose

The administered CM dose was significantly lower in LBW protocol group compared to fixed dose protocol group, both in terms of gI (36.2 ± 6.2 gI vs. 42.0 ± 0 gI, *p* < 0.001) and corresponding volume (103.4 ± 17.6 mL vs. 120.0 ± 0 mL, *p* < 0.001), as shown in Table 2. Within LBW protocol group, 7 patients (13.7%) received a CM dose greater than 120 mL (mean CM dose: 132.29 ± 8.34 mL, CM dose range: 124–144 mL).

### Objective image quality analysis

LBW protocol group accounted for significantly higher liver arterial CEI (23.02 ± 10.84 vs. 16.10 ± 9.65; *p* < 0.001), pancreatic arterial CNR (3.85 ± 2.04 vs. 2.92 ± 1.76; *p* = 0.004), pancreatic arterial CEI (66.45 ± 19.78 vs. 50.66 ± 17.29; *p* < 0.001), kidney arterial SNR (8.19 ± 2.61 vs. 7.38 ± 4.17; *p* = 0.016), and kidney arterial CNR (9.60 ± 3.55 vs. 7.90 ± 4.10; *p* = 0.003). Kidney CEI was superior in LBW protocol group in comparison with fixed dose group for both arterial (145.00 ± 42.53 vs. 122.17 ± 35.68; *p* = 0.002) and portal venous phase (103.56 ± 30.35 vs. 78.97 ± 58.75; *p* < 0.001).

Significantly lower portal vein SNR (9.33 ± 3.37 vs. 10.42 ± 3.01; *p* = 0.015) and CNR (7.57 ± 2.31 vs. 8.92 ± 3.31; *p* = 0.020) were observed in LBW protocol group compared to fixed dose group.

**Fig. 1:**
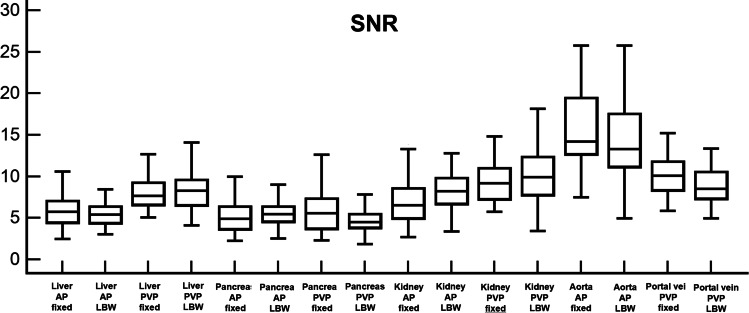
Box-and-whisker plots show average signal-to-noise ratios (SNR) of liver, pancreas, kidney, aorta, and portal vein on both arterial phase (AP) and portal venous phase (PVP), compared between fixed dose group (fixed) and lean body weight group (LBW). Boxes represent 25th and 75th percentile, horizontal lines 50th percentile (median), and whiskers minimum and maximum values

**Fig. 2:**
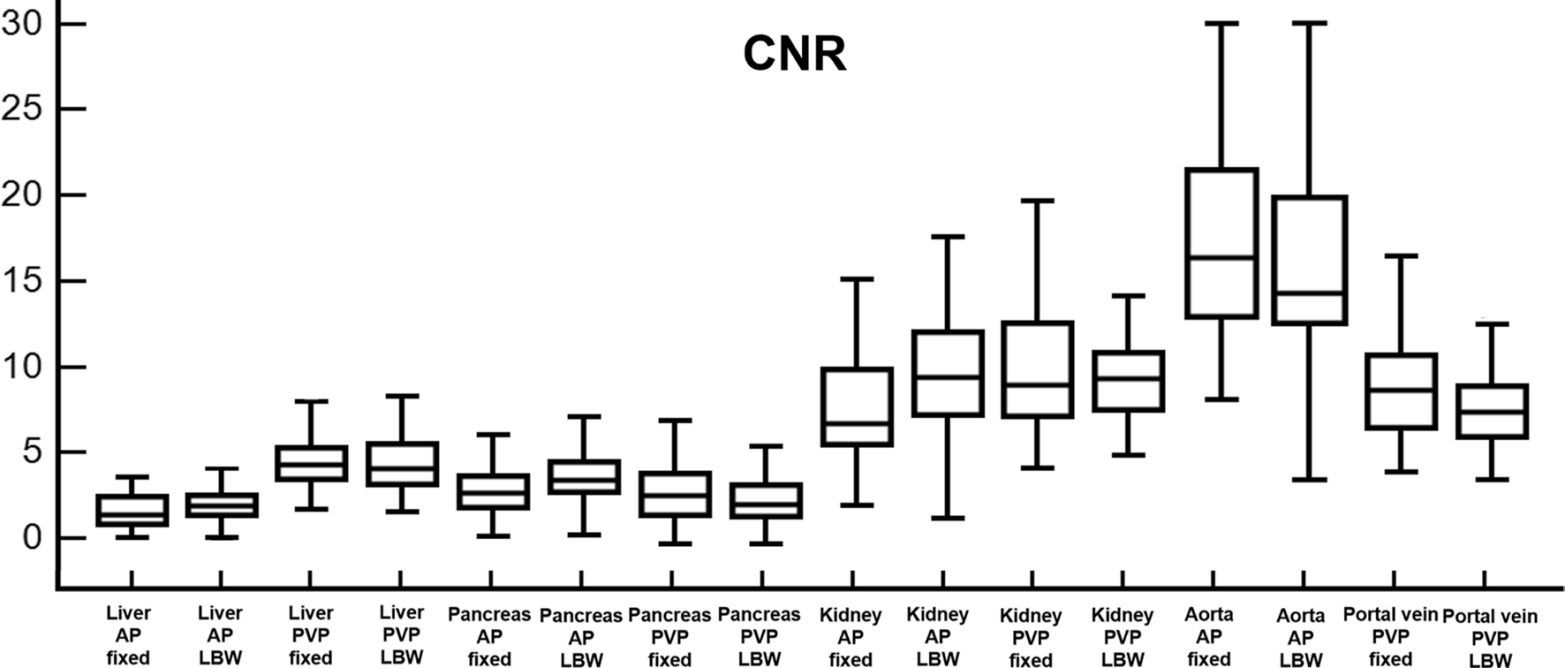
Box-and-whisker plots show average contrast-to-noise ratios (CNR) of liver, pancreas, kidney, aorta, and portal vein on both arterial phase (AP) and portal venous phase (PVP), compared between fixed dose group (fixed) and lean body weight group (LBW). Boxes represent 25th and 75th percentile, horizontal lines 50th percentile (median), and whiskers minimum and maximum values

**Fig. 3:**
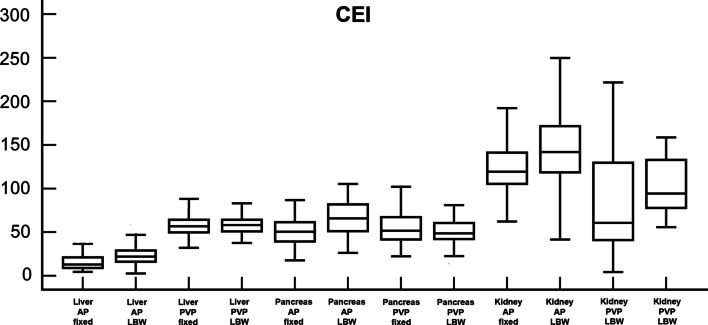
Box-and-whisker plots show average contrast enhancement index (CEI) of liver, pancreas, and kidney on both arterial phase (AP) and portal venous phase (PVP), compared between fixed dose group (fixed) and lean body weight group (LBW). Boxes represent 25th and 75th percentile, horizontal lines 50th percentile (median), and whiskers minimum and maximum values

No significant differences were observed in any of the remaining image quality parameters evaluated on both arterial and portal venous phases (all *p* ≥ 0.079). Results of objective image quality analysis are summarized in Table 3 and Figs. 1, 2, and 3.

**Table 3:** Results of objective image quality analysis

Parameter	Fixed dose protocol (*n* = 50)	LBW protocol (*n* = 50)	*p* value
Arterial phase			
Liver SNR	5.91 ± 1.88	5.58 ± 1.63	0.346
Liver CNR	1.72 ± 1.31	1.99 ± 1.27	0.123
Liver CEI, HU	16.10 ± 9.65	23.02 ± 10.84	< 0.001
Pancreas SNR	5.59 ± 3.55	5.90 ± 2.37	0.130
Pancreas CNR	2.92 ± 1.76	3.85 ± 2.04	0.004
Pancreas CEI, HU	50.66 ± 17.29	66.45 ± 19.78	< 0.001
Kidney SNR	7.38 ± 4.17	8.19 ± 2.61	0.016
Kidney CNR	7.90 ± 4.10	9.60 ± 3.55	0.003
Kidney CEI, HU	122.17 ± 35.68	145.00 ± 42.53	0.002
Aorta SNR	16.07 ± 5.75	14.56 ± 4.89	0.147
Aorta CNR	16.57 ± 6.42	17.47 ± 5.86	0.379
Portal venous phase			
Liver SNR	8.10 ± 1.96	8.22 ± 2.13	0.766
Liver CNR	4.48 ± 1.70	4.37 ± 1.61	0.730
Liver CEI, HU	59.61 ± 15.21	59.22 ± 11.14	0.796
Pancreas SNR	5.78 ± 2.37	4.91 ± 1.75	0.079
Pancreas CNR	2.69 ± 1.66	2.13 ± 1.30	0.065
Pancreas CEI, HU	55.15 ± 20.42	50.64 ± 13.86	0.220
Kidney SNR	9.54 ± 2.58	10.05 ± 3.10	0.370
Kidney CNR	9.95 ± 3.56	9.18 ± 2.21	0.198
Kidney CEI, HU	78.97 ± 58.75	103.56 ± 30.35	< 0.001
Portal vein SNR	10.42 ± 3.01	9.33 ± 3.37	0.015
Portal vein CNR	8.92 ± 3.31	7.57 ± 2.31	0.020

Data are means ± standard deviations

*LBW* lean body weight, *SNR* signal-to-noise ratio, *CNR* contrast-to-noise ratio, *CEI* contrast enhancement index, *HU* hounsfield unit

### Subjective image quality analysis

Detailed results are reported in Table 4. Subjective image quality assessment returned overall comparable results between the two group. LBW protocol group provided slightly higher contrast enhancement ratings compared to fixed dose protocol group for portal venous phase liver enhancement, arterial pancreatic enhancement, and both arterial and portal venous phase kidney enhancement, although all differences were not statistically significant (all *p* ≥ 0.213). No patients scored very poor or poor enhancement in both groups.

**Table 4:** Results of subjective image quality analysis

Parameter	Fixed dose protocol (*n* = 50)	Cohen’s *κ*	LBW protocol (*n* = 50)	Cohen’s *κ*	*p* value
Arterial hepatic enhancement	4 (2–5)	0.73 (95% CI 0.55–0.90)	4 (2–5)	0.71 (95% CI 0.54–0.88)	0.396
PVP hepatic enhancement	5 (3–5)	0.84 (95% CI 0.72–0.96)	5 (3–5)	0.80 (95% CI 0.60–1.00)	0.365
Arterial pancreatic enhancement	4 (2–5)	0.75 (95% CI 0.61–0.88)	4 (3–5)	0.88 (95% CI 0.71–1.00)	0.696
PVP pancreatic enhancement	4 (3–5)	0.90 (95% CI 0.80–1.00)	4 (3–5)	0.91 (95% CI 0.78–1.00)	0.659
Arterial renal enhancement	4 (3–5)	0.94 (95% CI 0.86–1.00)	4 (3–5)	0.97 (95% CI 0.91–1.00)	0.311
PVP renal enhancement	4 (3–5)	0.72 (95% CI 0.53–0.90)	4 (3–5)	0.94 (95% CI 0.86–1.00)	0.213

Data are medians with ranges in parentheses

*PVP* portal venous phase, *LBW* lean body weight

Overall inter-rater agreement was excellent for both fixed dose protocol (*κ* = 0.81 (95% CI 0.76–0.87)) and LBW protocol (*κ* = 0.87 (95% CI 0.81–0.93)) groups.

## Discussion

The aim of our study was to compare the performance of fixed-dose and LBW-adapted CM dosing protocols for multiphasic abdominal CT, in terms of image quality and parenchymal enhancement in oncologic patients. Our results showed that tailoring the CM volume according to LBW provides higher parenchymal enhancement of abdominal organs than using a fixed CM volume. Moreover, comparable or higher SNR and CNR were obtained in both arterial and portal venous phases despite the lower CM volume injected in the LBW tailored group.

Weight-adapted strategies tailored on patient’s TBW have been advocated for CM administration in multiphasic abdominopelvic CT, in order to overcome the drawbacks reported with fixed-dose protocols, namely the tendency to overestimation of CM amount in smaller patients or underdosage in heavier patients [5]. However, this technique may result in overeating of CM amount when applied to underweight individuals [25]. On the other end, adjusting the CM dose based on TBW may results in unnecessary elevated volumes of CM to be injected in overweight individuals, as a considerable fraction of their TBW is composed of adipose tissue, which has a negligible impact on solid organs’ enhancement [18, 26]. Parenchymal CM concentrations strongly depend on extracellular compartment volume since, after intravenous injection, CM promptly distributes from intravascular space to parenchymal extracellular space, without permeating into the intracellular space [2].

Among different body indexes, LBW has been demonstrated to better correlate with plasma and extracellular space volume [27, 28]. Therefore, dosing CM according to patients’ LBW accounted for differences in body composition and allowed to achieve consistent parenchymal enhancement even at reduced iodine load, by excluding from calculation of CM dose the irrelevant contribution of poorly perfused adipose tissue. Our results are in agreement with and corroborated those from previous studies that found optimal correlation between LBW and parenchymal enhancement, with improved per-patient uniformity [16, 19, 20].

Moreover, results obtained showed how CEI was higher in LBW protocol for liver and pancreas in arterial phase and for kidney in both arterial and portal venous phases. Despite these results might seem counterintuitive due to parenchymal enhancement dependency to iodine load, both CNR and SNR have no significant differences in liver, a solid organ usually considered for quantitative analysis [3, 5, 6, 9]. Regarding kidney and pancreas discrepancies, we hypothesized that measurements might be affected by the possible reduced dimension of kidney cortex while pancreas has not been studied in the specific late arterial pancreatic phase but in a late arterial phase tailored for multiphasic CT protocol for oncologic follow-up purpose.

Although it is beyond the scope of the study, we also observed that LBW-adapted protocol performed better in terms of parenchymal rather than vascular enhancement with reduced portal vein CNR and SNR in LBW-adapted protocol compared to fixed dose protocol. This result may be explained by the larger average CM volume administered to patients in the fixed dose protocol group. Indeed, vascular enhancement has been proven to be directly proportional to the injected CM volume, when injection rate and duration are maintained stable [2, 29]. Nonetheless, our LBW-adapted protocol is intended to be applied to multiphasic CT examinations in oncological setting, in which the main goal is tumor detection and response assessment rather than obtaining a robust angiographic study. Anyhow, mean aortic artery and portal vein attenuations in the LBW protocol group exceeded by far the minimal enhancement deemed diagnostic in discriminating between vessels and lymph nodes [30].

In our study, patients in the LBW-protocol group were given a significantly lower average amount of CM compared to those assigned to the fixed dose protocol. A reduced CM volume is especially beneficial for patients with cancer, who require multiple contrast-enhanced CT examinations to assess for disease progression and monitor the response to therapy. Moreover, it has been shown that oncologic patients are at high risk for developing acute kidney adverse events following iodinated CM administration, given the increased prevalence of associated risk factors, such as pre-existing kidney insufficiency, advanced age, dehydration, and concurrent nephrotoxic chemotherapeutic regimens [31, 32].

Lowering the average dose of administered CM should also be advisable in order to avoid unnecessary healthcare costs. Although a cost-effectiveness analysis was beyond the aim of our investigation, the potential cost savings achievable by the implementation of the present LBW-adapted CM dosing protocol are suggested by the results from previous studies that reported a remarkable cost reduction when a weight-adapted rather than a fixed-dose protocol is used [1, 7, 25].

There are several limitations of the present study that should be mentioned. First, as we assessed only a single type of CM (i.e. iodine concentration of 350 mgI/mL), additional studies would be required to demonstrate the reproducibility of our result when using CM with different iodine concentrations. Second, we solely evaluated the performance of LBW-adapted protocol in terms of image quality, further analysis to determine also the effects on lesions’ conspicuity and diagnostic accuracy was not investigated. Third, we calculated the LBW of each patient by using James formula; however, several prediction formulas for LBW have been previously reported and their application may yield results different from those observed in our study [27, 28]. However, the James formula is frequently used for estimating the CM dose and, although the use of Boer formula has been recommended in patients with a high BMI [33], no significant differences in objective image quality have been reported between these two formulas, when applied in a range of BMI as that observed in our population [14]. Furthermore, we determined the CM volume to be administered for LBW protocol group according to the calculated LBW rather than measured LBW, estimated by measuring patient fat body percentage with the aid of an analyzer scale [34]. Although the latter technique may have yielded a more accurate estimation, this approach would be technically impractical, while the use of calculated LBW supports the broad applicability of the present LBW-adapted protocol in the routine clinical practice. Lastly, although a lower iodine load achievable with LBW-adapted protocol could potentially reduce the risk of acute kidney failure in patients affected by renal chronic disease compared with a regular CM protocol, this aspect was not assessed in the study.

## Conclusions

In conclusion, the results of this prospective randomized multicenter study demonstrate that dosing CM according to patient’s LBW rather than administering a fixed dose of CM allows for a significant reduction of the injected CM volume with no detrimental effects on image quality and parenchymal enhancement. Implementation of an LBW-adapted protocol should be considered in oncologic patients in order to reduce costs and minimize the risks of contrast-induced acute kidney adverse events.

## Data Availability

The datasets used and/or analysed during the current study are available from the corresponding author on reasonable request.

## Abbreviations

BMI: Body mass index
BSA: Body surface area
CEI: Contrast enhancement index
CM: Contrast media
CNR: Contrast-to-noise ratio
CT: Computed tomography
LBW: Lean body weight
ROI: Region-of-interest
SNR: Signal-to-noise ratio
